# Receptors for Insulin-Like Growth Factor-2 and Androgens as Therapeutic Targets in Triple-Negative Breast Cancer

**DOI:** 10.3390/ijms18112305

**Published:** 2017-11-02

**Authors:** Nalo Hamilton, David Austin, Diana Márquez-Garbán, Rudy Sanchez, Brittney Chau, Kay Foos, Yanyuan Wu, Jaydutt Vadgama, Richard Pietras

**Affiliations:** 1 UCLA School of Nursing, University of California at Los Angeles, Los Angeles, CA 90095, USA; 2 UCLA Jonsson Comprehensive Cancer Center, University of California at Los Angeles, Los Angeles, CA 90095, USA; dmarquez@mednet.ucla.edu (D.M.-G.); jayvadgama@cdrewu.edu (J.V.); 3 Department of Medicine, Division of Cancer Research and Training, Charles Drew University School of Medicine and Science, Los Angeles, CA 90059, USA; davidaustin@cdrewu.edu (D.A.); yanyuanwu@cdrewu.edu (Y.W.); rpietras@mednet.ucla.edu (R.P.); 4 UCLA David Geffen School of Medicine, Department of Medicine, Division of Hematology-Oncology, University of California at Los Angeles, Los Angeles, CA 90095, USA; 5 Department of Biology, California State University Channel Islands, Camarillo, CA 93012, USA; rudy.sanchez917@myci.csuci.edu; 6 Department of Integrative Ecology and Evolutionary Biology and Physiology, UCLA College of Life Sciences, University of California at Los Angeles, Los Angeles, CA 90095, USA; chaubrittney@gmail.com; 7 Department Physiological, UCLA College of Life Sciences, University of California at Los Angeles, Los Angeles, CA 90095, USA; kaymaliafoos@yahoo.com

**Keywords:** triple-negative breast cancer, insulin-like growth factor-2, androgen receptor, BMS-754807, NVP-AEW541, enzalutamide, IGF1R/IR inhibition, IGF2 signaling, AKT kinase, apoptosis

## Abstract

Triple-negative breast cancer (TNBC) occurs in 10–15% of all breast cancer patients, yet it accounts for about half of all breast cancer deaths. There is an urgent need to identify new antitumor targets to provide additional treatment options for patients afflicted with this aggressive disease. Preclinical evidence suggests a critical role for insulin-like growth factor-2 (IGF2) and androgen receptor (AR) in regulating TNBC progression. To advance this work, a panel of TNBC cell lines was investigated with all cell lines showing significant expression of IGF2. Treatment with IGF2 stimulated cell proliferation in vitro (*p* < 0.05). Importantly, combination treatments with IGF1R inhibitors BMS-754807 and NVP-AEW541 elicited significant inhibition of TNBC cell proliferation (*p* < 0.001). Based on Annexin-V binding assays, BMS-754807, NVP-AEW541 and enzalutamide induced TNBC cell death (*p* < 0.005). Additionally, combination of enzalutamide with BMS-754807 or NVP-AEW541 exerted significant reductions in TNBC proliferation even in cells with low AR expression (*p* < 0.001). Notably, NVP-AEW541 and BMS-754807 reduced AR levels in BT549 TNBC cells. These results provide evidence that IGF2 promotes TNBC cell viability and proliferation, while inhibition of IGF1R/IR and AR pathways contribute to blockade of TNBC proliferation and promotion of apoptosis in vitro.

## 1. Introduction

Breast cancer (BC) is the second leading cause of cancer death among women [1,2,3]. Most patients with BC have tumors that express estrogen receptor-α (ERα-66kD) and/or progesterone receptor (PR) at diagnosis and therefore are candidates for endocrine treatments to improve clinical outcomes. However, triple-negative breast cancers (TNBC) are a heterogeneous subtype of BCs that lack nuclear expression of estrogen receptor-α (ERα-66kD) and progesterone receptor (PR) and have no overexpression of HER2 receptors [4,5]. Consequently, targeted endocrine and HER2-directed therapies are not recommended for management of TNBCs. Although TNBCs account for 15–20% of all BC cases [1], TNBCs account for almost half of all BC deaths [1,4,6,7]. TNBCs are generally larger in size, initially of higher grade, exhibit lymph node involvement at diagnosis and are biologically more aggressive than other BC subtypes [8]. Women diagnosed with TNBC have shorter overall survival and a higher risk of both local and distant recurrence, and metastases are more likely to occur in the brain and lungs rather than bone as compared to other breast cancers [8]. Women diagnosed with TNBC experience higher rates of recurrence [7,9]. Identification of novel therapeutic approaches for TNBC is critical because the median survival for women with metastatic TNBC is currently less than 12 months, and virtually all women with metastatic TNBC ultimately die of their disease despite systemic cytotoxic chemotherapy.

The progression of TNBC remains to be fully understood. The identification of novel targets in the proliferative process in TNBC could ultimately help to improve the outcomes of patients diagnosed with this deadly disease. Current evidence indicates that insulin/insulin-like growth factor (IGF) signaling pathways play a significant role in BC progression [10,11,12,13,14]. The circulating regulatory hormones of this system include insulin, insulin-like growth factor-1 (IGF1) and insulin-like growth factor-2 (IGF2). These circulating growth hormones are reported to enhance TNBC progression [12,15,16,17], and the bioavailability in vivo of both IGF1 and IGF2 are modulated by six different IGF-binding proteins (IGFBPs) [18,19,20].

Importantly, IGF2 is highly expressed in archival TNBC samples from women in the clinic [21]. IGF2 binding to IGF1R/IR-A in vitro is reported to facilitate downstream signaling for TNBC cell survival that is mediated in part by modulating the activity of estrogen receptor-beta (ERβ) which differs from the classical ERα [12,21]. Normally, circulating IGF2 is bound by mannose-6 phosphate/IGF2 receptor (IGF2R) and IGFBPs, predominantly by IGFBP-6 [20,22]. However, IGF2 also binds to tumor cell surface IGF1R and IR, with preference for IR isoform A (IR-A). IGF1R and IR are both receptor tyrosine kinases that are expressed in TNBC cells, with evidence of IGF1R amplification in some archival TNBC specimens obtained after neoadjuvant treatments in the clinic [23]. The activation of IGF1R and IR heterodimers stimulates downstream effectors in the mitogen-activated protein kinase (MAPK) and/or phosphatidylinositol 3-kinase (PI3K) AKT/protein kinase B (PKB) pathways resulting in tumor progression [19]. Although early phase clinical trials raised hope for the use of IGF1R-specific antibodies for cancer therapy, results from phase II–III trials of these agents in unselected patients have not demonstrated significant benefits in the clinic [24]. At this time, further investigations are focusing on specific signaling pathways that may prove effective in the clinic [24,25]. To date, limited investigation has focused on pathways stimulated by IGF2 in TNBC progression and the therapeutic potential of targeting identified pathways to improve patient outcomes. Further clinical interventions may benefit from discovery of alternative strategies to target this critical pathway and the identification of predictive biomarkers to select patients as candidates for IGF-targeted therapies.

The androgen receptor (AR), a member of the nuclear steroid hormone receptor superfamily, is reported to be expressed in most invasive breast cancers when ER and PR are not detectable, as well as in 17–35% of established TNBC specimens [26,27,28,29,30]. Although the exact role of ARs in TNBC remains to be elucidated, current research indicates that AR is highly conserved throughout TNBC progression and impacts tumor proliferation [28,29,31]. Inhibition of AR signaling significantly reduces TNBC cell proliferation, migration, invasion and increases apoptosis in vitro, while significant reduction of tumor viability is reported by AR inhibition in vivo [32,33]. TNBCs with high levels of AR expression are also reported to correlate with poor clinical outcomes [29]. Additionally, independent work shows that AR overexpression in tamoxifen-resistant breast tumors results in constitutive activation of IGF1R and PI3K/AKT pathways [34].

Systemic chemotherapy is currently the standard of care for neoadjuvant and/or adjuvant treatment of patients with TNBC, yet recurrence rates and mortality remain high in women with this aggressive disease [9,35]. Apart from early clinical trial data indicating antitumor efficacy of immune checkpoint inhibitors in a minority of patients with TNBC [36,37], targeted treatment options for women with TNBC are currently not available. Thus, the identification of biologic targets that block the progression of TNBCs could lead to reduced recurrence of this deadly disease, thereby leading to improved patient outcomes. Due to their altered expression and potential dysregulated downstream signaling pathways in TNBC, assessing interactions of IGF and AR pathways in this disease may help to better understand the underlying processes of TNBC progression, as well as provide novel targets to ultimately improve patient survival. To this end, we investigated the antitumor effect of inhibiting the IGF1R/IR and AR pathways alone and in combination in a panel of established TNBC cell lines. Results of this preclinical study suggest that inhibition of IGF1R//IR and AR pathways significantly reduce TNBC progression and stimulate TNBC cell death.

## 2. Results

### 2.1. IGF2 Promotes the Viability of TNBC Cells

**Figure 1:**
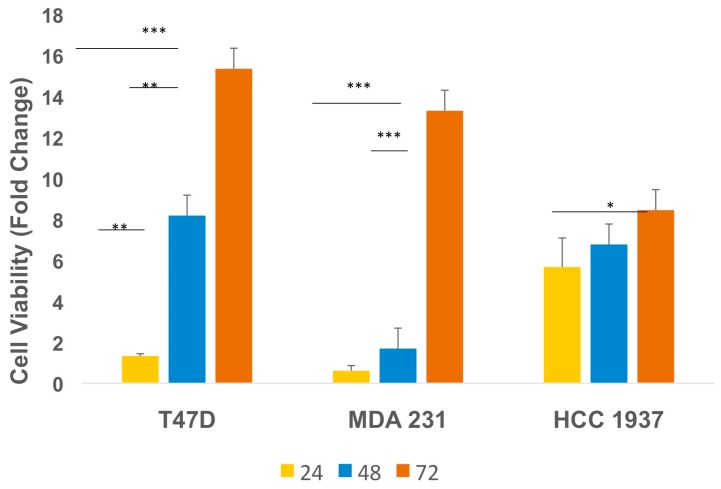
Insulin-like growth factor-2 (IGF2) promotes triple-negative breast cancer (TNBC) cell viability. Initial plating consisted of 1.0 × 10^5^ cells per well. Plated cells were cultured in complete media for 48 h followed by incubation in serum- and phenol red-free medium for 24 h. Cultures were then maintained in IGF2 (100 ng/mL)-containing media for 24, 48 and 72 h. IGF2 culture media was refreshed every 24 h. Viable cells were counted using trypan blue exclusion. Data represents at least three independent experiments performed in duplicate. *** *p* ≤ 0.0002; ** *p* ≤ 0.007; * *p* ≤ 0.05. Error bars represent standard deviation. T47D (ERα+/PR+ BC cell line) used as a positive control.

Although IGF2 is highly expressed in archival TNBC specimens from the clinic [21], direct effects of IGF2 on TNBC cell proliferation and/or viability remain to be fully elucidated. This clinical data raises the hypothesis that IGF2 may have the ability, in part, to promote tumor growth via autocrine and/or paracrine mechanisms. To assess the effect of IGF2 treatment on cell viability and proliferation, we first exposed TNBC cells to IGF2 (100 ng/mL) using serum-free and phenol red-free media. In these in vitro studies at 72 h post-treatment, IGF2 significantly promoted cell proliferation in T47D (ERα+/ERβ+) cells, as well as in TNBC MDA-MB-231 and HCC 1937 cells (Figure 1; *p* < 0.05), and the action is significantly different from that in control cells.

These results suggest that IGF2 may play an important role, in part, in maintaining TNBC viability and proliferative activity.

### 2.2. IGF2 Treatment Impacts Downstream TNBC Signaling Molecules

**Figure 2:**
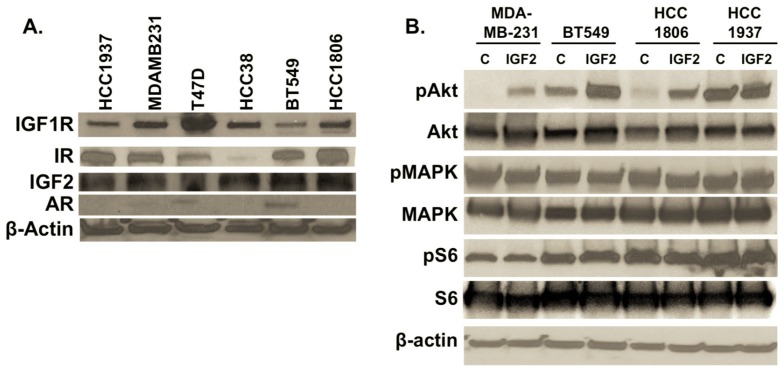
(**A**) Expression of IGF2, IGF1R, insulin receptor (IR) and androgen receptor (AR) in TNBC cultures. Total protein was isolated from cell cultures. Forty micrograms of protein were separated and transferred to PVDF membranes for detection of IGF1R (1:500, Cell Signaling #3027, Danvers, MA, USA), IR (1:500, Cell Signaling #3025), IGF2 (1:1000, AbCam ab9574), and AR (1:500, Cell Signaling #5153). β-actin (1:2000, Sigma #A1978, St. Louis, MO, USA) was used as a loading control. TNBC cells include HCC1937, MDA-MB-231, HCC38, BT549 and HCC1806, with ERα-/PR-positive T47D cell line as a control; (**B**) Effects of IGF2 treatment on downstream phosphorylation of MAPK, AKT and S6. IGF2-induced activation of IGF1R leads to increased phosphorylation of AKT in most TNBC cells assessed. TNBC cultures were treated with IGF2 (100 ng/mL) in serum- and phenol red-free media for 20 min. Total protein was isolated, separated and transferred to PVDF membranes. Detection of MAPK (1:1000; Cell Signaling #9102), pMAPK (Cell Signaling #4370), S6 (1:2000; Cell Signaling #2217), pS6 (Cell Signaling #4858), AKT (1:1000 Cell Signaling, #4685) and pAKT (Cell Signaling #4060) was accomplished following the manufacturer’s recommended protocols (Methods). C = control vehicle-treated cells. IGF2 = cells treated with IGF2 for 20 min. Western immunoblots are representative of three independent experiments.

In view of previous reports on the potential relationship between IGF1R and AR signaling pathways [19,32,34], we investigated our panel of TNBC cell lines for the presence of IGF1R signaling mediators and AR. In each TNBC cell line, IGF2 as well as IGF1R and IR are detected in varying amounts (Figure 2A, Lanes 1–6).

As previously reported [32,38,39], AR is readily detected in T47D (ERα+/ERβ+/PR+) and TNBC BT549 (ER-/PR-/HER-) cells, with minimal levels in MDA-MB-231 (ER-/PR-/HER2-) cells (Figure 2A).

It is reported that stimulation of IGF2 binding to IGF1R/IR receptors activates downstream signaling by MAPK and/or AKT signaling pathways [40]. In TNBC cells exposed to IGF2 for 20 min, we note that phosphorylation of MAPK is similar between control and IGF2-treated TNBC cells, with minimal effects on S6 phosphorylation, a downstream mediator of the mTOR signaling pathway (Figure 2B). However, notable phosphorylation of AKT occurs in MDA-MB-231, BT549 and HCC 1806 cell lines (Figure 2B).

**Figure 3:**
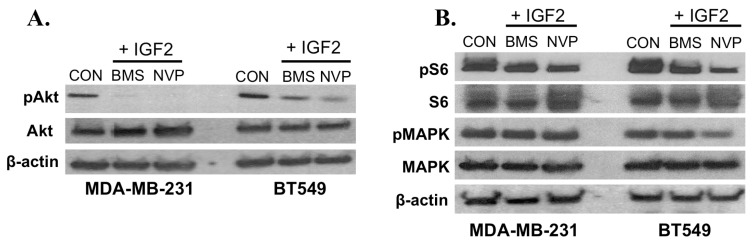
Inhibitory effects of BMS-754807 and NVP-AEW541 on IGF2-induced signaling. (**A**) IGF1R/IR Inhibitors reduce pAKT. TNBC cell cultures were serum starved for 3 h, then incubated with BMS-754807 or NVP-AEW541 for 1 h. IGF2 (100 ng/mL) was added to the serum-free, and phenol red-free media for 20 min. Total protein was isolated, separated and transferred to PVDF membranes. Detection of Akt (1:1000; Cell Signaling #4685), pAkt (1:1000, Cell Signaling #4060) were evaluated using western immunoblot analysis; (**B**) IGF1R/IR antagonists slightly reduce pS6 but not pMAPK in TNBC cells. TNBC cultures were serum starved, exposed to IGF1R/IR inhibitors followed by IGF2. The effects of this treatment on MAPK (1:1000, Cell Signaling #9102), pMAPK (1:1000, Cell Signaling #4370), S6 (1:2000, Cell Signaling #2217), and pS6 (1:2000, Cell Signaling #4858) was done as per manufacturer’s recommended protocols (Methods). CON = control vehicle-treated cells. BMS + IGF2 = cells treated with BMS-754807 and IGF2. NVP + IGF2 = cells treated with NVP-AEW541 + IGF2. Western immunoblots are representative of at least three independent experiments.

A number of studies have investigated the effect of IGF1R and IR inhibitors on ERα+ tumor cell proliferation and growth [6,13,19]. To assess the potential benefit of treatment with two clinically-distinct receptor inhibitors on IGF2 signaling, we treated TNBC cultures with the dual IGF1R/IR tyrosine kinase inhibitor BMS-754807, and IGF1R inhibitor NVP-AEW541, followed by IGF2 stimulation. Notably, levels of IGF2-induced pAKT and pS6 are both diminished by combination treatments with BMS-754807 and NVP-AEW541 (Figure 3), suggesting that IGF2-mediated effects on phosphorylation of AKT and S6 occur at least in part via IGF1R/IR receptor pathways (Figure 3).

### 2.3. Inhibition of TNBC Proliferation and Induction of Cell Death by IGF1R/IR and AR Antagonists

**Figure 4:**
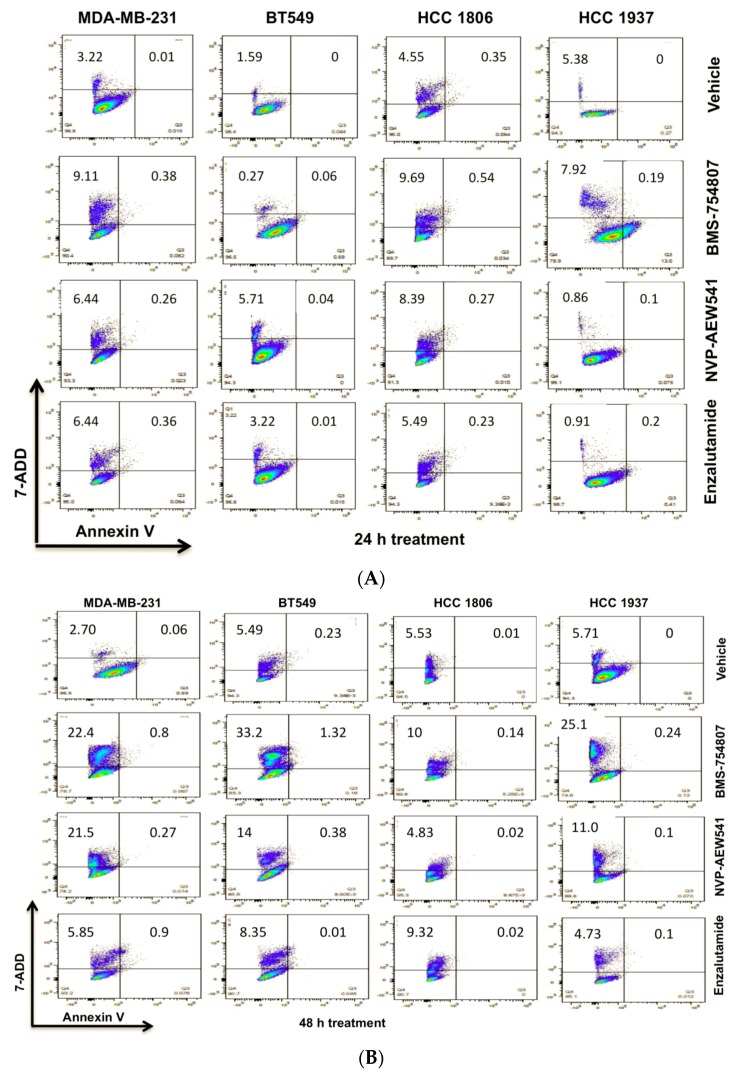
IGF1R/IR antagonists induce TNBC cell death. TNBC cells were grown to 75–80% confluence in complete media, then transferred to the indicated inhibitor-conditioned media for 24 h (**A**) or 48 h (**B**). Cells were harvested and prepared as per the manufacturer’s recommended protocol for flow cytometry using 7-AAD and Annexin-V. Analyses were done using LSRII and FloJo Software.

Using flow cytometry, we assessed the ability of BMS-754807 and NVP-AEW541, along with enzalutamide, an AR inhibitor, to induce TNBC cell death at 24 and 48 h post-treatment (Figure 4A,B).

**Figure 5:**
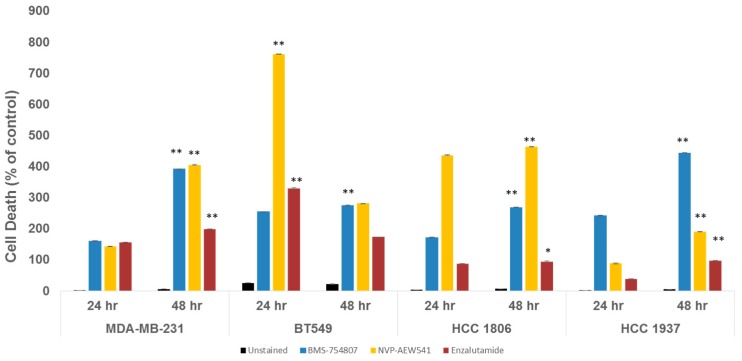
TNBC cell death is promoted by IGF1R/IR and AR antagonists. Results show % cell death of vehicle-treated controls. An independent control was used for each cell line. TNBC cells were exposed to either BMS-754807 (20 μM), NVP-AEW541 (8 μM) or enzalutamide (20 μM) (*n* = 3 independent experiments). Error bars represent standard deviation (SD). Data was analyzed by Prism GraphPad software using an unpaired Student *t*-test. *p*-Value < 0.05 are considered significant. ** *p* ≤ 0.005; * *p* ≤ 0.05.

As compared to 24 h post-treatment, MDA-MB-231 cells exhibited enhanced apoptosis in response to BMS-754807, NVP-AEW541 and enzalutamide at 48 h (see Figure 4 and Figure 5; with *p* < 0.0001). For BT549 cells, cell death was induced by BMS-754807 at 48 h. However, in this TNBC cell line with robust AR expression, treatment with enzalutamide and NVP-AEW541 as single agents significantly enhanced cell death at 24 h (Figure 5; *p* < 0.005).

Interestingly, HCC 1806 and HCC 1937 cultures, which are cells without detectable AR (Figure 2), significantly responded to BMS-754807, NVP-AEW541 and enzalutamide (Figure 5, *p* < 0.005). In this panel of TNBC cell lines, inhibition by BMS-754807 consistently induced cell death. These data suggest that inhibitors targeting IGF1R/IR homo- or heterodimers can prevent TNBC tumor growth. Additionally, cultures with low to undetectable AR expression responded to enzalutamide. As reported by Baron et al., our results support inhibitory effects of enzalutamide in non-luminal AR (LAR) TNBC cell subtypes (32; MDA-MB-231, BT549, HCC 1806).

**Figure 6:**
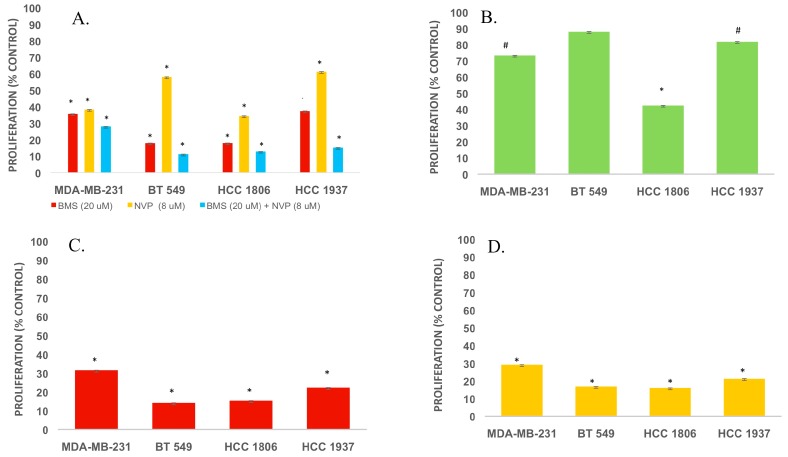
(**A**–**D**) Effects of dual therapy with IGF1R/IR and AR antagonists on TNBC proliferation. TNBC cells were grown in 96-well plates to 50–60% confluence in complete media. (**A**) Cells were maintained in phenol red-free RMPI 1640 media supplemented with 2% cFBS and either NVP-AEW541 (8 μM, IGF1R inhibitor; Selleckchem) or BMS-754807 (20 μM, IGF1R/InsR inhibitor; Selleckchem), alone or in combination; (**B**) Cells were exposed to media containing enzalutamide (20 μM, AR inhibitor; Selleckchem) and proliferation was assessed after 72 h; (**C**) Cells were cultured in media containing BMS-754807 and enzalutamide for 72 h or (**D**) in media containing NVP-AEW754 and enzalutamide for 72 h. Thereafter, cell proliferation was measured using the Celltiter 96^®^ Aqueous Proliferation Assay (Promega, Madison, WI, USA). # *p*-value < 0.05, * *p*-value < 0.001. SD: standard deviation (*n* = 3).

In view of emerging evidence for growth factor receptor-steroid hormone receptor cross-communication in malignancy [41], and the reduction of TNBC proliferation and viability [42,43,44], we evaluated BMS-754807, NVP-AEW541 and enzalutamide alone and in selected combinations on TNBC proliferation (Figure 6).

Combination treatment with BMS-754807 and NVP-AEW541 significantly reduced TNBC cell proliferation to a greater extent than administration of either BMS-754807 or NVP-AEW541 alone (Figure 6A; *p* < 0.001). Among HCC 1806 cells with minimal to undetectable AR expression, the AR antagonist enzalutamide significantly reduced cell proliferation (Figure 6B; *p* < 0.001). In contrast, enzalutamide elicited a minor reduction of BT549 cell proliferation, yet these cells exhibit more robust expression of AR (Figure 2 and Figure 6B).

Notably, cell proliferation was further reduced in TNBC cells of the non-luminal androgen receptor subtype when enzalutamide was used in combination with either BMS-754807 or NVP-AEW541 (Figure 6C,D; *p* < 0.001). Thus, combination therapy targeting IGF1R/IR and AR may have some further benefit in suppressing TNBC.

### 2.4. BMS-754807 and NVP-AEW541 Alter AR Expression in Mesenchyma-Subtype TNBC Cells

**Figure 7:**
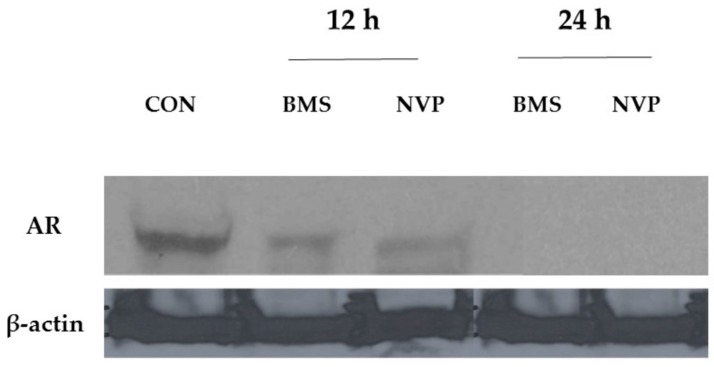
Effect of IGF1R/IR antagonists on AR expression in mesenchymal-subtype TNBC cell line BT549. BT549 (mesenchymal-like) cultures were exposed to control (CON), BMS-754807 (BMS; 20 μM) or NVP-AEW807 (NVP; 8 μM) for 12 or 24 h. Total protein was isolated, processed and transferred to PVDF membranes which were probed for AR expression (1:500, Cell Signaling #5153). β-actin (1:2000, Sigma-Adlrich #A1978) was used as a loading control. Western immunoblot representative of three independent experiments.

To find if the reduction of TNBC proliferation in response to combined treatments with either BMS-754807 or NVP-AEW541 may also affect AR expression (Figure 7), we assessed AR levels in BT549 cells that express a robust amount of AR under baseline conditions (Figure 2, Lane 5).

Cells were exposed to both IGF1R inhibitors for 12 or 24 h. At 12 h, the results indicate that NVP-AEW541, an antagonist that prevents auto-phosphorylation of IGF1R, reduces expression levels of AR to a greater extent than BMS-754807, a dual IGF1R/IR inhibitor (Figure 7). Notably at 24 h both inhibitors reduce AR expression to virtually undetectable levels by Western immunoblot.

## 3. Discussion

Apart from the administration of neoadjuvant and/or adjuvant cytotoxic chemotherapies, there are currently limited therapeutic options for the majority of patients afflicted with advanced TNBC. Emerging reports from early clinical trials suggest that anti-PD-1 or anti-PD-L1 immune checkpoint inhibitors may have clinical benefit in very limited numbers of TNBC patients [36,37,45]. However, other targeted therapies that have proven effective for patients with ERα-positive or HER2-overexpressing subtypes of breast cancer are not available for TNBC due to the lack of ERα and PR expression and HER2 amplification, thus leaving most women with TNBCs without targeted treatment options. To further investigate potential biologic targets in TNBC to improve patient outcomes, we assessed the interplay between the IGF2 and AR signaling pathways in TNBC proliferation.

TNBC is a heterogeneous disease that can be classified into distinct molecular subtypes by gene expression profiling [46]. Currently, there are four TNBC subtypes, basal-like 1 (BL1), basal-like 2 (BL2), mesenchymal-like (M), and luminal androgen receptor expression (LAR), that demonstrate differences in diagnosis, age, local and distant disease progression, histopathology and in the response to similar neoadjuvant chemotherapy that may inform in the selection of optimal chemotherapeutic regimens [47]. The majority of all TNBCs are basal-like, with mesenchymal-like more common than LAR [48]. We find that both BL and M subtypes in particular, as represented by established TNBC cell lines, respond to IGF1R/IR and AR antagonists alone and in combination treatments. Our preclinical studies indicate that IGF1R- and AR-targeted therapies reduce TNBC cell proliferation and induce cell death, indicating that these receptors may serve as attractive targets for TNBC treatment going forward.

Enzalutamide is an androgen receptor antagonist which prevents androgen receptor nuclear translocation, coactivator recruitment and DNA binding. Although studies present mixed patient outcomes related to high and low AR expression [29,49,50,51,52,53], several preclinical studies have evaluated the therapeutic potential of AR inhibition in TNBC progression [5,54,55]. Treatment of TNBC cell lines with enzalutamide significantly reduces TNBC proliferation, invasion and amphiregulin growth factor secretion [32]. Although it was hypothesized that treatment with enzalutamide would only be effective in the LAR molecular subtype of TNBC, other molecular subtypes including basal-like and mesenchymal-like subtypes were also responsive to antiandrogen interventions [5], outcomes that are supported by this work. Additionally, clinical trials indicate that enzalutamide, alone or in combination, is well tolerated in women with advanced breast cancer [55,56,57].

Due to the molecular heterogeneity and high rate of drug resistance among TNBCs, preclinical studies are investigating the targeting of multiple signaling pathways to stop TNBC proliferation [58]. These dual therapies have proven to be more effective in preventing TNBC proliferation compared to single drug therapies [42,44,58]. In line with this notion, we investigated the effect of BMS-754807 and NVP-AEW541, two mono-therapies tested previously as single agents in breast cancer clinical trials [59,60]. Our preclinical study indicates a potential benefit of BMP-754807 and NVP-AEW541 administered together and in combination with enzalutamide to stimulate TNBC cell death and reduce TNBC proliferation. Furthermore, our experiments indicate that more profound inhibition of TNBC cell proliferation can be achieved by the use of these drugs in combination therapies in vitro (Figure 4). Further studies will be needed to determine whether drug combinations determined in this work are synergistic or additive [61,62]. Additionally, the tolerability and safety of these combinations in vivo remains to be assessed.

Importantly, the lack of clinical efficacy of IGF-targeted therapies may well be a result of the lack of predictive biomarkers to select those patients most likely to benefit from these therapeutics. Several preclinical studies have investigated the correlation between patient treatment outcomes with tumors bearing a specific molecular background [23,47,63]. For example, patients with TNBC or KRAS-mutant lung cancers may obtain optimal benefit from IGF1R inhibitor drugs [42,64,65]. Other reports suggest that genomic alterations in PI3K/AKT and IGF1R may be actionable targets in TNBC [23]. However, the challenge in unlocking the potential for IGF-targeted therapies lies in defining predictive biomarkers [23,47]. In prior studies, we documented increased expression of IGF2 in tumorous and in neighboring non-tumorous stromal and epithelial cells in archival breast tissue from women with TNBC [21]. These observations implicate IGF2 in the promotion of TNBC growth via autocrine and/or paracrine mechanisms. Independent investigators have also identified IGF2 as a key factor in the genesis of breast tumor spheres [66]. The IGF2 receptor, IGF1R, is expressed at high levels in cancer stem cell (CSC)-enriched populations in primary breast cancers. Moreover, IGF2-PI3K/AKT signaling induced expression of a stemness transcription factor and IGF2 itself. Notably, treatment with anti-IGF1/2 antibodies blocked tumorigenesis derived from the IGF1R^high^ CSC-enriched population in a patient-derived xenograft model [66]. Similarly, AR is necessary for anchorage independence and CSC circumvention of anoikis, processes that are inhibited by enzalutamide [67]. Our findings, as presented here, indicate a benefit in TNBC outcomes by inhibiting IGF2 activity using targeted therapies against IGF1R/IR and AR to prevent tumor growth and potentially metastasis.

## 4. Materials and Methods

### 4.1. Cell Culture

Cell lines were obtained from the American Type Culture Collection (ATCC) and cultured according to ATCC recommendations. Briefly, T47D (ERα+/ERβ+) and triple negative (ERα-negative, PR-negative, HER2-negative) cell lines HCC 1806, MDA-231, HCC 38, HCC 1937, and BT549 [21] were cultured in RPMI 1640 media (ATCC) and with 10% fetal bovine serum (FBS; Hyclone, Logan, UT, USA), 100,000 units penicillin and 100,000 units streptomycin sulfate (Cellgro, Manassas, VA, USA). Cultures were maintained at 37 °C in a 5% CO_2_ atmosphere using a tissue culture incubator. For estrogen-free conditions, cells were maintained in phenol red-free RPMI media with dextran-coated, charcoal-treated (DCC) FBS (cFBS).

### 4.2. Cell Viability and Proliferation Assays

Assessment of IGF2 effects on TNBC viability was performed using 1.0 × 10^5^ cells/well seeded in a 6-well Transwell plate (Corning, Tewksbury, MA, USA) in complete media. After 24 h in estrogen-free conditions, cell cultures were exposed to phenol red-free, estrogen-free RPMI media containing IGF2 (100 ng/mL; Sigma-Aldrich, St. Louis, MO, USA) for 24, 48 or 72 h. IGF2-containing medium was replaced daily. At the indicated time points, cell numbers and cell viability were assessed using a Cell Counter (BioRad, Hercules, CA, USA) and trypan blue dye-exclusion assays, respectively.

To assess the roles of IGF1R, IR, and AR on TNBC proliferation, 5 × 10^3^ cells per well were plated in 96-well plates. Cells were allowed to grow to 50–60% confluence in RPMI 1640 media containing 10% FBS. Media for test drugs were composed of phenol red-free RMPI 1640 media supplemented with 2% charcoal-dextran stripped FBS (Gemini BioProducts, West Sacramento, CA, USA) containing IGF1R inhibitor NVP-AEW541 (8 μM, Selleckchem, Houston, TX, USA), IGF1R/InsR inhibitor BMS-754807 (20 μM, Selleckchem), or AR inhibitor enzalutamide (20 μM, Selleckchem), alone or in selected combinations. Cell cultures were maintained in the conditioned media for 72 h. Cell proliferation was then assessed using the Celltiter-96^®^ Aqueous Proliferation Assay (Promega, Madison, WI, USA) following the manufacturer’s recommended protocol.

### 4.3. Assays of Cell Viability and Apoptosis/Cell Death by Flow Cytometry

For flow cytometry experiments, cells were plated and grown to 80% confluence in complete media. Cultures were then exposed to the IGF1R inhibitor NVP-AEW541 (8 μM, Selleckchem), IGF1R/IR inhibitor BMS-754807 (20 μM, Selleckchem), or AR inhibitor enzalutamide (20 μM, Selleckchem), in media containing phenol red-free RMPI 1640 media supplemented with 2% charcoal-dextran-stripped FBS (Gemini BioProducts, CA, USA) for 24 or 48 h. As per the manufacturer’s recommended protocol, cells were then removed and stained with 7-AAD and Annexin-V (Affymetrix, Inc., San Diego, CA, USA) for viability and apoptosis assays, respectively. Cells were examined using an LSR II Benchtop Analyzer (BD Biosciences, San Jose, CA, USA) with FlowJo Collector’s Edition software (FloJo, LLC, Ashland, OR, USA).

### 4.4. Inhibition Assays

To assess the inhibitory effect of BMS-754807 and NVP-AEW541 on IGF2-stimulated signaling, TNBC cells were grown to 75–80% confluence in complete media, then cultured for 3 h in serum-free, and phenol red-free RPMI 1640 media. BMS-754807 (20 μM) or NVP-AEW541 (8 μM) was added to the serum-free, and phenol red-free media for 1 h followed by the addition of IGF2 (100 ng/mL) for 20 min.

### 4.5. Gel Electrophoresis and Immunoblotting

To assess the expression of IGF1R, IR, and AR, TNBC cultures were maintained as described above. Using RIPA buffer, total protein was isolated, and protein concentration was determined using the Bradford assay. Forty micrograms of total cell protein were resolved by 4–15% SDS-PAGE, transferred to a PVDF membrane and probed with antibodies directed against IGF1R (1:500, Cell Signaling #3027), IR (1:500, Cell Signaling #3025), IGF2 (1:1000, AbCam ab9574), pAkt (1:1000, Cell Signaling #4060), MAPK (1:1000, Cell Signaling #9102), pMAPK (1:1000, Cell Signaling #4370), S6 (1:2000, Cell Signaling #2217), pS6 (1:2000, Cell Signaling #4858), and androgen receptor AR (1:500, Cell Signaling #5153). β-actin (1:2000, Sigma-Aldrich #A1978) was used as a loading control.

### 4.6. Statistical Analysis

GraphPad Prism software (Available online: www.graphpad.com) was used for statistical analysis. Student’s *t*-test was used to determine significance as appropriate. *p*-Value < 0.05 were considered significantly different.

## 5. Conclusions

New insights made from preclinical and correlative biologic studies could well lead to the determination of predictive TNBC biomarkers that may impact clinical decision-making and provide new avenues for mechanistic exploration leading to clinical application. Hence, our experiments on novel dual therapies targeting IGF2 and AR signaling pathways hold promise as potential therapeutic targets for TNBC treatment going forward. Indeed, the results of emerging studies indicate that TNBC proliferation and survival are dependent on IGF1R/IR, and that their inhibition leads to reductions in proliferation and increments in cell death [68]. Molecular targeting therapies that include anti-IGF1R antibodies, anti-IGF1/IGF2 antibodies, and small molecule inhibitors that suppress IGF1R and IR kinase activity as well as AR antagonists deserve further evaluation in the potential management of TNBC.

## Abbreviations

IGF1R	Insulin-like growth factor-1 receptor
AR	Androgen receptor
TNBC	Triple-negative breast cancer
IGF2	Insulin-like growth factor-2
BC	Breast cancer
PR	Progesterone receptor
IGF1	Insulin-like growth factor-1
IGF	Insulin-like growth factor
IGFBPs	IGF-binding proteins
ERβ	Estrogen receptor-β
IGF2R	IGF2 receptor
IR	Insulin receptor
