# Correction: Hamilton et al. Receptors for Insulin-Like Growth Factor-2 and Androgens as Therapeutic Targets in Triple-Negative Breast Cancer. *Int. J. Mol. Sci.* 2017, *18*, 2305

**DOI:** 10.3390/ijms25052579

**Published:** 2024-02-23

**Authors:** Nalo Hamilton, David Austin, Diana Márquez-Garbán, Rudy Sanchez, Brittney Chau, Kay Foos, Yanyuan Wu, Jaydutt Vadgama, Richard Pietras

**Affiliations:** 1 UCLA School of Nursing, University of California at Los Angeles, Los Angeles, CA 90095, USA; 2 UCLA Jonsson Comprehensive Cancer Center, University of California at Los Angeles, Los Angeles, CA 90095, USA; dmarquez@mednet.ucla.edu (D.M.-G.); jayvadgama@cdrewu.edu (J.V.); 3 Department of Medicine, Division of Cancer Research and Training, Charles Drew University School of Medicine and Science, Los Angeles, CA 90059, USA; davidaustin@cdrewu.edu (D.A.); yanyuanwu@cdrewu.edu (Y.W.); rpietras@mednet.ucla.edu (R.P.); 4 UCLA David Geffen School of Medicine, Department of Medicine, Division of Hematology-Oncology, University of California at Los Angeles, Los Angeles, CA 90095, USA; 5 Department of Biology, California State University Channel Islands, Camarillo, CA 93012, USA; rudy.sanchez917@myci.csuci.edu; 6 Department of Integrative Ecology and Evolutionary Biology and Physiology, UCLA College of Life Sciences, University of California at Los Angeles, Los Angeles, CA 90095, USA; chaubrittney@gmail.com; 7 Department Physiological, UCLA College of Life Sciences, University of California at Los Angeles, Los Angeles, CA 90095, USA; kaymaliafoos@yahoo.com

In the original publication [1], there were mistakes in Figure 4A and Figure 7 as published. In Figure 4A, there is an apparent duplication of flow cytometry data for the 24 h timepoint. The image for the MDA-MB-231 vehicle is duplicated for BT549 enzalutamide. In the original manuscript, Figure 7 was the result of parallel blots developed on film. As such, concerns were raised regarding the image quality and splicing of loading control.

In light of these concerns, the experiment was repeated, with additional controls, and all samples were run together. Due to the inclusion of new controls, additional information is needed for the legend associated with Figure 7. The corrected images for Figure 4A and Figure 7 along with the updated legend for Figure 7 are presented below.

The authors state that the scientific conclusions are unaffected. This correction was approved by the Academic Editor. The original publication has also been updated.

## Figures and Tables

**Figure 4:**
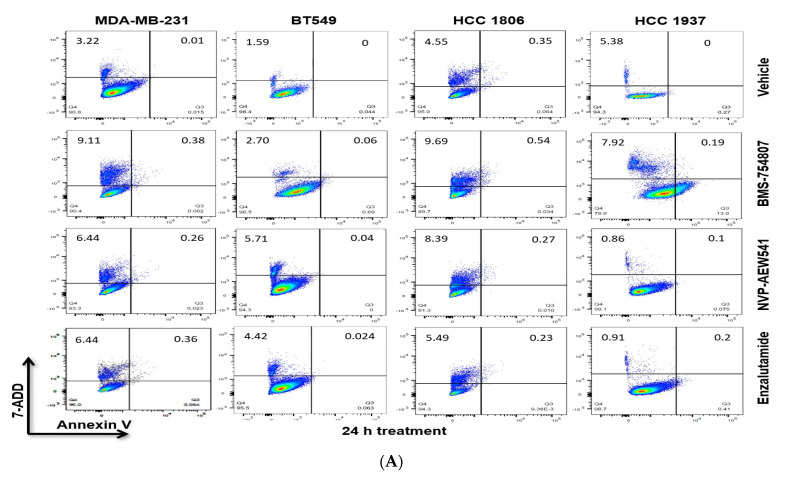

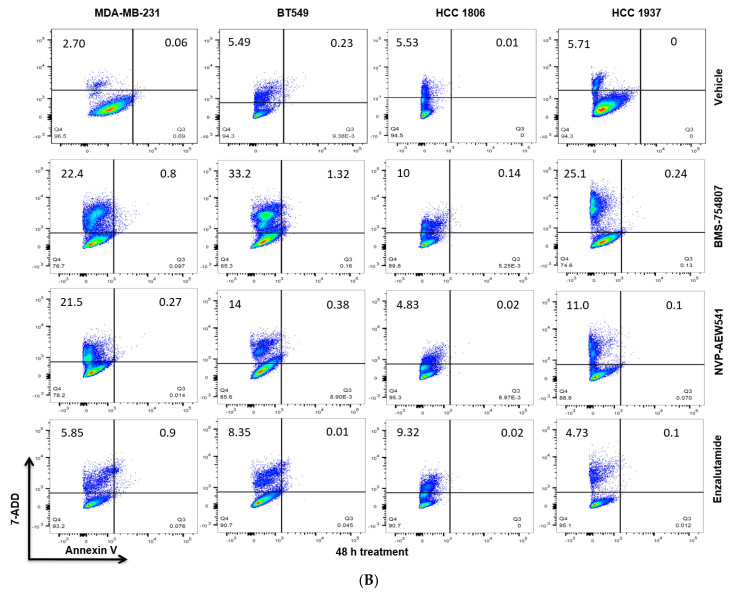
IGF1R/IR antagonists induce TNBC cell death. TNBC cells were grown to 75–80% confluence in complete media, then transferred to the indicated inhibitor-conditioned media for 24 h (**A**) or 48 h (**B**). Cells were harvested and prepared as per the manufacturer’s recommended protocol for flow cytometry using 7-AAD and Annexin-V. Analyses were performed using LSRII and FloJo Software (BD FACSDiva Software v8.0.3).

**Figure 7:**
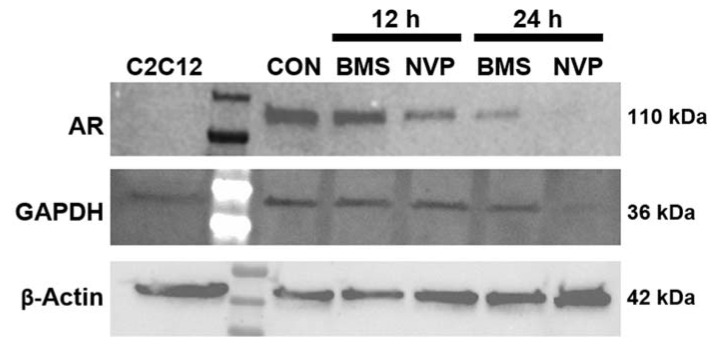
Effect of IGF1R/IR antagonists on AR expression in mesenchymal-subtype TNBC cell line BT549. BT549 (mesenchymal-like) cultures were exposed to control media (CON), BMS-754807 (BMS; 20 μM) or NVP-AEW807 (NVP; 8 μM) containing media for 12 or 24 h. Total protein was isolated, processed and transferred to PVDF membranes which were probed for AR expression (1:500, Cell Signaling #5153, Cell Signaling Technology, Danvers, MA, USA). GAPH (1:1000; h-FAB Rhodamine, BioRad, Hercules, CA, USA) and β-actin (1:500, Santa Cruz Biotechnology, Inc., Dallas, TX, USA) were used as loading controls. C2C12, a subclone of the murine myoblast cell line, was used as a negative control for AR specificity. Western immunoblot was representative of four independent experiments.
